# Understanding Variations Among Individuals With Child Sexual Exploitation Material Offences: A Cumulative Meta-Analysis

**DOI:** 10.5964/sotrap.16273

**Published:** 2026-05-08

**Authors:** Serra Baskurt, Melissa O’Donaghy, Kelly M. Babchishin

**Affiliations:** 1 Department of Psychology, Carleton University, Ottawa, ON, Canada; Centre for Criminology (Kriminologische Zentralstelle – KrimZ), Wiesbaden, Germany

**Keywords:** CSEM, child sexual abuse, cumulative meta-analysis

## Abstract

Understanding the characteristics of individuals involved in child sexual exploitation material (CSEM) offences is crucial for policy, treatment, and case prioritization. This study examined the extent to which 238 men adjudicated for CSEM-exclusive offences (sexual history is limited to CSEM offences; *M_age_* = 41.9, *SD* = 13.2) differ from 94 men with mixed offences (both CSEM and contact sexual offences; *M_age_* = 43.4, *SD* = 13.9; median assessment year = 2009). We conducted a cumulative meta-analysis by integrating our findings with the latest meta-analysis on CSEM characteristics (i.e., Babchishin et al., 2015). Of the 10 characteristics examined, four showed significant deviation from the meta-analytical averages. Mixed offending individuals showed higher levels of prior offences (*d_weighted_* = .44, 95% CI [.34, .53], *Q*_Δ_ = 18.35, *p* < .001), emotional identification with children (*d_weighted_* = .28, 95% CI [.15, .40], *Q*_Δ_ = 18.35, *p* < .001), and empathy deficits (*d_weighted_* = .26, 95% CI [.13, .38], *Q*_Δ_ = 29.05, *p* < .001) compared to CSEM-exclusive individuals, with differences increasing when incorporating our new sample. Conversely, the difference in prior violent offences decreased (*d_weighted_* = .78, 95% CI [.64, .92], *Q*_Δ_ = 9.06, *p* = .028), with mixed individuals showing a greater reduction than the CSEM-exclusive group. No differences were noted for age, education, victim access, prior sexual offences, impulsivity, and substance use. This study highlights the distinct risk profiles of mixed versus CSEM-exclusive groups, underscoring the need for differentiated management approaches.

## Abstract (plain language summary)

**Background:**

Individuals engaged in Child Sexual Exploitation Material (CSEM; also legally referred to as child pornography in the United States) offences demonstrate a heterogenous profile.

**Why was this study done?:**

Recent studies highlight some shifts in characteristics among individuals with CSEM offences. This study was conducted to update our understanding of the changing characteristics among individuals with CSEM offences, building on the findings from the latest meta-analysis by Babchishin et al. (2015).

**What did the researchers do and find?:**

From a sample of 332 men adjudicated for CSEM offences, we divided our sample into two groups: CSEM-exclusive, comprising of 238 men whose sexual offending exclusively involved CSEM offences, and mixed, comprising of 94 men with both CSEM and contact sexual offences. We examined the characteristics of both groups and compared our findings with those from Babchishin et al.’s (2015) meta-analysis. The difference between the two groups increased in terms of prior offences, emotional identification with children, and empathy deficits, with individuals in the mixed offending group scoring higher on all of these characteristics than those in the CSEM-exclusive group. These trends indicate that mixed offending individuals continue to pose a consistently higher risk than CSEM-exclusive individuals.

**What do these findings mean?:**

The findings highlight the need for tailored intervention strategies, as mixed-offending individuals demonstrate a higher risk profile compared to those with CSEM-exclusive offences.

## Highlights

- Mixed offending individuals (who commit both CSEM and contact sexual offences) exhibit a higher risk profile for sexual recidivism compared to CSEM-exclusive offending individuals (who exclusively commit CSEM offences as part of their sexual offending), even in more contemporaneous samples (median assessment year = 2009).
- Mixed offending individuals show more empathy deficits, impulsivity, and emotional identification with children than CSEM-exclusive individuals; a difference that has increased since the former meta-analysis.
- Continued research is essential to index the typical profiles of CSEM-offending individuals and to formulate effective management strategies.

Individuals engaged in Child Sexual Exploitation Material (CSEM) offences demonstrate a heterogenous profile. CSEM-exclusive individuals are characterized by their exclusive involvement in CSEM offences, whereas mixed offending individuals engage in both CSEM and contact sexual offences as part of their sexual offending history. Although the same offending behaviours do not characterize both groups, they share some similarities. For instance, both offending groups tend to be convicted at similar ages, typically in their early forties (e.g., Baskurt et al., 2025; Long et al., 2013; McCarthy, 2010) and are predominantly White (e.g., Aslan & Edelmann, 2014; Babchishin et al., 2015).

There are also notable distinctions across both groups. Meta-analytic findings indicate that mixed offending individuals have a more extensive criminal history, characterized by more prior offences (*d_fixed-effect_* = 0.35 [with an outlier removed], *N* = 1,422, *k*[studies] = 7), greater access to victims (*d_fixed-effect_* = 0.32, *N* = 2,309, *k* = 9), higher levels of sexual interest in children (*d_fixed-effect_* = 0.50, *N* = 1,205, *k* = 6), and heightened cognitive distortions (*d_fixed-effect_* = 0.31, *N* = 1,175, *k* = 5) compared to individuals whose sexual offences are exclusively CSEM offences (Babchishin et al., 2015).

## Characteristics of Individuals With CSEM Offences

Recent research suggests that the profile of individuals engaged in CSEM offending may be subject to variation across studies, with some indicating patterns such as a rise in pedophilic interests and a decline in substance use compared to earlier cohorts (Christensen & Tsagaris, 2020; Henshaw et al., 2018). For example, Christensen and Tsagaris’ (2020) content analysis of sentencing remarks (*N* = 29) found a considerably higher rate of unemployment (32%) and incomplete schooling (43%) within CSEM-exclusive offending individuals, in comparison to previous studies (14.7% and 10.6%, respectively; Babchishin et al., 2011). In contrast, an earlier study by Aslan and Edelmann (2014) found that individuals in the mixed (*n* = 38) and CSEM-exclusive (*n* = 74) offending groups had similar rates of stable employment (63% and 61%, respectively; *Cohen’s h* = 0.04, *p* = .81). The authors did however observe a slight difference in retirement status, where individuals in the mixed group were slightly more likely to be retired (9%) compared to those in the CSEM-exclusive group (5%), although this difference was not statistically significant (*Cohen’s h* = 0.16, *p* = .44).

Notable differences were also observed in terms of CSEM content, such that a considerably large proportion of cases (nearly 80%) involved materials depicting very young children (e.g., under the age of three; Christensen & Tsagaris, 2020). This heightened prevalence of young children considerably differs from earlier findings, where such instances were observed in a smaller fraction (19%) of cases (Wolak et al., 2005), suggesting a potential increase in pedophilic interests among CSEM offending individuals. Further, differences in image consumption showed that a slightly higher proportion of mixed individuals (62%) accessed the most extreme images characterized by violence and sadistic sex compared to 52% among CSEM-exclusive individuals (Aslan & Edelmann, 2014).

Along the same lines, Soldino et al. (2019) examined the characteristics of men arrested for CSEM offences in Spain, distinguishing between those with CSEM-exclusive offences (*n* = 283) and a very small group of those with mixed offences (*n* = 18). Pedophilic interests were operationalized as scores of three or higher on the Correlates of Admission of Sexual Interest in Children (CASIC; Seto & Eke, 2017). Although no statistically significant difference in pedophilic interests was found between the two offending groups, individuals involved in mixed offending displayed pedophilic interests at more than twice the rate of CSEM-exclusive individuals (*M_CSEM-E_* = 8.1%, *M_mixed_* = 22.2%, *V* = .13, *p* = .22). Similarly, Soldino et al. (2021) also did not identify any significant group difference between CSEM-exclusive (*n* = 255) and mixed individuals (*n* = 49) in terms of pedophilic interests as measured by the CASIC (*M_CSEM-E_* = 9.4%, *M_mixed_* = 16.3%, odds ratio [*OR*] = 0.47, *p* > .05; Seto & Eke, 2017), although mixed offending individuals still reported more pedophilic interests than those with CSEM-exclusive offences. On the other hand, Babchishin et al.’s (2015) meta-analysis identified a significant group difference in pedophilia between CSEM-exclusive and mixed individuals, with mixed individuals exhibiting more pedophilia (*d_fixed-effect_* = 0.50 [with outlier removed], *N* = 1,205, *k* = 6). In addition to pedophilic interest, Kuhle et al. (2017) found that sexual preoccupation appears to be associated with mixed offending in general. Individuals in the mixed offending group displayed significantly greater sexual preoccupation than those in the CSEM-exclusive group (*OR* = 1.69 versus *OR* = 1.40; both *p* < .001), suggesting that elevated sexual preoccupation could play an important role in the development of mixed offending pathways.

Marital status patterns also appear to vary between groups. Babchishin et al.’s (2015) meta-analysis reported a significant difference between both offending groups, where mixed offending individuals were less likely to have never been married than CSEM-exclusive individuals (*d_fixed-effect_* = 0.16 [with an outlier removed], *N* = 945, *k* = 4). More recently, Soldino et al. (2019) found that 283 men with CSEM-exclusive offences were almost twice as likely to have never been married than 18 men with mixed offences, although this difference did not reach statistical significance (*M_CSEM-E_* = 45.2%, *M_mixed_* = 27.8%, *V* = .06, *p* = .70). Similarly, Soldino et al. (2021) found no significant difference in the rate of never being married between CSEM-exclusive and mixed offending individuals (*M_CSEM-E_* = 45.1%, *M_mixed_* = 42.9%, *OR* = 0.90, *p* > .05), suggesting that the relationship between marital status and the type of CSEM offending may be less pronounced than previously reported.

Substance use patterns show similar variability, as noted by Henshaw et al. (2018), who highlighted differing trends in substance use behaviours between CSEM-exclusive (*n* = 456) and mixed groups (*n* = 256). In contrast to Babchishin et al. (2015) who found that mixed offending individuals reported greater substance use (*d_fixed-effect_* = 0.35, *N* = 1,143, *k* = 5), Henshaw et al. (2018) reported a smaller difference between both groups (*M_CSEM-E_* = 6.14%, *M_mixed_* = 7.42%, Cohen’s *h* = -0.06). Similar findings were reported by Aslan and Edelmann (2014), who found that 31% of CSEM-exclusive individuals and 21% of mixed offending individuals had a documented history of substance misuse, a difference that was not statically significant (*Cohen’s h* = 0.23, *p* = .11). The minimal difference in substance use between the two offending groups suggests an increasing similarity between them.

The profiles of individuals involved in both mixed and CSEM-exclusive offences may be evolving due to broader societal changes (e.g., COVID-19 pandemic) and technological advancements (McMahan et al., 2024; Savage, 2024; Steel et al., 2024), such as the darknet (Gannon et al., 2023). The expansion of internet access and technological advancements has facilitated easier access to CSEM, with a 290% increase from 2014 to 2022 in Canada (Savage, 2024). These shifts have not only changed how CSEM is accessed and distributed but may also be influencing the profiles of individuals who engage in these offences. Research suggests that increased digital connectivity can alter offending pathways by making it easier for individuals with limited prior criminal histories to access CSEM anonymously and with minimal risk of detection (Kloess & van der Bruggen, 2023; Seto, 2025). This accessibility may attract a broader and more heterogeneous group of offenders, potentially blurring the distinctions between CSEM-exclusive and mixed offending individuals. For example, some individuals who previously may not have committed contact offences might now have more opportunity and reinforcement through online communities that normalize such behaviour. As a result, the lines between CSEM-exclusive and mixed offending may be shifting, calling for closer examination of offender subtypes in more recent contexts. Such findings underscore that the characteristics of individuals with CSEM offences may differ depending on sample composition, location, and assessment period. The present study aims to refine and extend these earlier findings through a cumulative meta-analysis by integrating a large, independent sample. This approach enables a more precise estimation of group differences between CSEM-exclusive and mixed individuals, providing clarity on patterns that may transcend individual study variability.

## Current Study

In this study, we used a cumulative meta-analysis to build on and extend the findings of Babchishin et al. (2015). Our goal was to examine whether the addition of a new, independent sample drawn from individuals adjudicated for CSEM offences in British Columbia meaningfully shifts the overall patterns observed in the existing literature. We combined our findings from 332 CSEM offending men (*N_CSEM-E_* = 238, *N_mixed_* = 94) with Babchishin et al.’s (2015) meta-analysis to (a) assess whether our findings significantly differ from the meta-analysis average, and (b) calculate a new weighted meta-analytical average on factors related to offending behaviour including internet demographics, antisociality, and general psychological profiles.

The selection of risk factors such as prior offences, emotional identification with children, impulsivity, substance use, and empathy deficits are grounded in established models and empirical research on sexual offending (e.g., Mann et al., 2010; Seto, 2019; Seto et al., 2023). More specifically, Seto’s Motivational-Facilitation Model (Seto, 2019) provides a useful framework, positing that sexual offending behaviour results from a combination of motivational factors (e.g., sexual interest in children, emotional congruence with children) and facilitative factors (e.g., impulsivity, substance use, poor self-regulation). For example, emotional identification with children reflects a motivational factor that may drive CSEM or contact offending, while impulsivity and substance use act as facilitators by reducing inhibitory control (Seto, 2019). Prior offending histories are strong predictors of both CSEM and contact sexual recidivism (Baskurt et al., 2025), and empathy deficits are recognized as contributing to the maintenance of atypical behaviour by allowing individuals to minimize the harm caused to victims (Bartels & Merdian, 2016).

## Method

### Participants

The current study included 332 men adjudicated for CSEM offences that were under community supervision in British Columbia and received either a STABLE-2007 or ACUTE-2007 assessment between January 1, 2005, and June 4, 2013 (*Mdn* year = 2009). While our sample overlaps with Babchishin et al. (2023), it is slightly larger because our study did not require sexual recidivism information. As per Hanson et al. (2016), our sample can be classified as a routine/complete sample, representing the population of men adjudicated for sexual offences. The current sample included 238 men with CSEM-exclusive offences (*M_age_* = 41.90, *SD* = 13.19) and 94 men with mixed offences (*M_age_* = 43.45, *SD* = 13.88). From the dataset of men being supervised by B.C. Corrections for a sexual offence, we created two CSEM offending groups based on STABLE items and criminal history information, following the same coding rules as Babchishin et al. (2023). Specifically, we excluded participants scored solely on the STABLE-2000 version of the scale, as it was unclear whether their victims were adults or children. For the mixed offending group, we included men with CSEM offences who also had at least one child victim from a contact sexual offence. Additionally, we removed individuals from the mixed group if they: (1) did not have any “deviant” victims as defined by the STABLE-2007 criteria; (2) had no score on the emotional congruence with children item from the STABLE-2007 scale (since this item is not scored if there are no victims under 14 years old); or (3) had only non-contact or non-solicitation sexual charges or convictions. Our CSEM-exclusive group was defined as individuals with a CSEM sexual offence and no history of contact sexual offences. Descriptive statistics for the sample are presented in Table 1.

**Table 1:** Sample Characteristics

Variable	CSEM-exclusive	Mixed
**Age, *M* (*SD*; *n*)**	41.90 (13.19; 238)	43.45 (13.88; 94)
Race/ethnicity, *% (n / N)*		
Asian	2.6% (6 / 233)	1.1% (1 / 94)
Black	0.4% (1 / 233)	0% (0 / 94)
White	90.1% (210 / 233)	86.2% (81 / 94)
East Indian	0% (0 / 233)	2.1% (2 / 94)
Hispanic	0.4% (1 / 233)	0% (0 / 94)
Indigenous	3.0% (7 / 233)	6.4% (6 / 94)
Metis	0% (0 / 233)	1.1% (1 / 94)
Other	3.4% (8 / 233)	3.2% (3 / 94)
Education, *% (n / N)*		
None	0.5% (1 / 215)	0% (0 / 91)
Elementary	1.4% (3 / 215)	3.3% (3 / 91)
Grade 7, 8, 9	4.7% (10 / 215)	9.9% (9 / 91)
Grade 10, 11	17.7% (38 / 215)	23.1% (21 / 91)
Grade 12	37.2% (80 / 215)	34.1% (31 / 91)
Vocational	13.5% (29 / 215)	15.4% (14 / 91)
University	25.1% (54 / 215)	14.3% (13 / 91)
**Total sex offences^a^ *M (SD; n)***	1.1 (0.3; 238)	2.1 (2.4; 94)
Recidivism rate, % (*n* / *N; f / u*^b^)		
Any sexual	3.8 (9 / 238; 4.1)	7.6 (7 / 94; 3.6)
Any contact sexual	0.4 (1 / 238; 4.2)	4.3 (4 / 94; 3.8)
Any CSEM offending	3.4 (8 / 238; 4.1)	3.2 (3 / 94; 3.8)

*Note.* CSEM = Child Sexual Exploitation Materials.

^a^Includes prior charges or convictions. ^b^Average follow-up time in years.

### Measures

We examined variables including age, education level, victim access, prior offences (any, sex, and violent), emotional identification with children, impulsivity, substance use, and empathy deficits. Demographic information was obtained from an administrative data pull of B.C. Corrections. Items of risk tools (i.e., ACUTE-2007 and STABLE-2007; Brankley et al., 2017, 2019) were scored by correctional officers as part of routine case management procedures.

***Demographic Variables:*** Age at release was recorded, and education level response options included none, elementary, Grade 7, 8, 9, Grade 10, 11, Grade 12, vocational, and university. Prior offences were defined by any prior charge(s) or conviction(s) (any, sexual, and violent including contact sexual offence).

***Emotional Identification With Children***, as measured by the STABLE-2007 item, is operationalized as a preference for spending time with children rather than with adults, as well as age-inappropriate interests and activities. This item is only applicable for individuals who have committed at least one sexual offence against a victim under 14 years old and measured on a three-point scale: 0 indicating “no problem”, 1 indicating “slight problem”, and 2 “definite problem” (Fernandez et al., 2014).

***Empathy Deficits***, as measured by the STABLE-2007 item, is defined as a lack of concern for others that is evident through the individual’s lifestyle and behaviours. This item is scored on a three-point scale (Fernandez et al., 2014).

***Impulsivity*,** as measured by the STABLE-2007 item, is defined as the extent to which an individual displays impulsive behaviour across various settings (e.g., personal relationships, financial, and leisure) and this item is scored on a three-point scale (Fernandez et al., 2014).

***Substance Use***, as measured by the ACUTE-2007 item, is defined as the use of prohibited and illegal substances (alcohol/drug use) and this item is scored on a four-point scale: 0 indicating “not present”, 1 indicating “maybe present”, 2 indicating “present”, and 3 “intervene now” (Fernandez et al., 2015).

***Victim Access***, as measured by the ACUTE-2007 item, assesses the extent to which an individual increases their risk by seeking contact with or gaining access to potential victims (Fernandez et al., 2015). This item is scored on a four-point scale: 0 indicating “little to no opportunity to meet and/or interact with potential victims”, 1 indicating “incidental contact with a potential victim that appears unintentional”, 2 indicating “creating or taking advantage of an opportunity to interact with preferred victim group”, and 3 indicating “intentional access to victims” (Fernandez et al., 2015).

### Statistical Analyses

#### Cumulative Meta-Analysis

We used Cohen’s *d* as an effect size to compare CSEM-exclusive and mixed groups on available variables. Consistent with Babchishin et al. (2015), a positive *d* value indicated that mixed-offending individuals exhibited more risk factors (e.g., emotional identification with children) than CSEM-exclusive individuals. Effect sizes were computed and summarized using the Cumulative Meta-Analysis method outlined by Hanson and Broom (2005). Specifically, the new cumulative effect size, ${\bar{E}}_{\text{new}}$ was computed using the following formula:

$${\bar{E}}_{\text{new}}=\frac{\left(\frac{E_{k}}{v_{k}}+\frac{{\bar{E}}_{\text{old}}}{v_{\text{old}}}\right)}{\left(\frac{1}{v_{k}}+\frac{1}{v_{\text{old}}}\right)}$$

Here, *E*_k_ and *v*_k_ represent the effect size and its variance observed in the current study (referred to as study “*k*”), respectively, while ${\bar{E}}_{\text{old}}$ represents the fixed cumulative effect size computed (as per Babchishin et al., 2015) before the current study. To determine whether our study and Babchishin et al.’s (2015) study are from the same population and to compare our findings with their 2015 meta-analysis, we used the *Q* statistic method:

$$Q_{\Delta }=\frac{{\left({\bar{E}}_{\text{old}}-{\bar{E}}_{\text{new}}\right)}^{2}}{v_{\text{old}}}+\frac{{\left(E_{k}-{\bar{E}}_{\text{new}}\right)}^{2}}{v_{k}}$$

The change in variability (*Q*_Δ_) follows a chi-squared (χ^2^) distribution with one degree of freedom. We reported the fixed-effects model for all comparisons. All analyses were double run by the first and second authors using R Studio syntax (R Studio Team, 2020), which can be found in the Supplementary Materials.

## Results

### Indicators of Sexual Offending and Demographics

Table 2 provides the effect sizes from the previous meta-analysis (Babchishin et al., 2015) and the current study. Access to victim showed a significant change, with a *d_weighted_* of .34 (95% CI [.26, .42]). The current study indicates that the difference between the mixed and CSEM-exclusive groups has increased (*d_currentstudy_* = .58, 95% CI [.32, .85]) compared to the previous meta-analysis, which primarily focused on access to children (*d_oldmeta_* = .32, 95% CI [.21, .43]).

**Table 2:** Comparing Individuals With CSEM-Exclusive Offences to Individuals With Mixed Offences on Demographic and Behavioural Characteristics

Variable	Babchishin et al. (2015)		Current study		Cumulative Meta-analysis					
	*d*	[95% CI]	*d*	[95% CI]	*d_weighted_*	[95% CI]	*k*	*Q*_Δ_	*p*	*Q*
Younger	-0.04	[-0.13, 0.04]	-0.12	[-0.35, 0.12]	-0.05	[-0.12, 0.03]	14	0.39	1.000	15.76
Low education	0.10	[-0.04, 0.24]	**0.31**	[0.06, 0.56]	**0.14**	[0.03, 0.24]	10	2.32	.985	13.67
Victim access	**0.32**	[0.21, 0.43]	**0.58**	[0.32, 0.85]	**0.34**	[0.26, 0.42]	10	3.42	.905	**26.07**
Any prior offences^a^	**0.35**	[0.21, 0.49]	**0.92**	[0.68, 1.17]	**0.44**	[0.34, 0.53]	8	**17.37**	**.026**	**29.69**
Prior sex offences^a^	**1.12**	[0.90, 1.35]	**1.41**	[1.15, 1.67]	**1.16**	[1.06, 1.26]	8	4.03	.855	**21.43**
Prior violent offences^a^	**0.94**	[0.61, 1.28]	**0.48**	[0.24, 0.72]	**0.78**	[0.64, 0.92]	4	**9.06**	**.028**	**10.14**
Emotional ID	0.15	[-0.01, 0.32]	**0.86**	[0.53, 1.18]	**0.28**	[0.15, 0.40]	4	**18.35**	**< .001**	**18.55**
Impulsivity	0.02	[-0.14, 0.18]	**0.47**	[0.21, 0.73]	0.11	[-0.003, 0.23]	5	9.26	.052	**13.85**
Substance use	**0.35**	[0.19, 0.50]	0.20	[-0.06, 0.46]	**0.32**	[0.22, 0.43]	6	1.04	.904	3.02
Empathy deficits	0.08	[-0.06, 0.23]	**0.90**	[0.63, 1.16]	**0.26**	[0.13, 0.38]	4	**29.05**	**< .001**	**36.12**

*Note.* CSEM = Child Sexual Exploitation Materials; ID = identification. Fixed effect meta-analytical average effect sizes are presented. A positive *d* indicates that individuals with mixed offences had more characteristics that were risk-relevant than those with CSEM-exclusive offences. *d_weighted_* = *d* derived from fixed-effect meta-analysis. *Q*_Δ_ shows if the effect size significantly changes with the current study added to the old study, whereas *Q* represents the heterogeneity of the current study. Bolded values are significant at *p* < .05.

^a^Includes prior charges or convictions. Babchishin et al. (2015) outlier removed effect sizes are presented.

**Figure 1:**
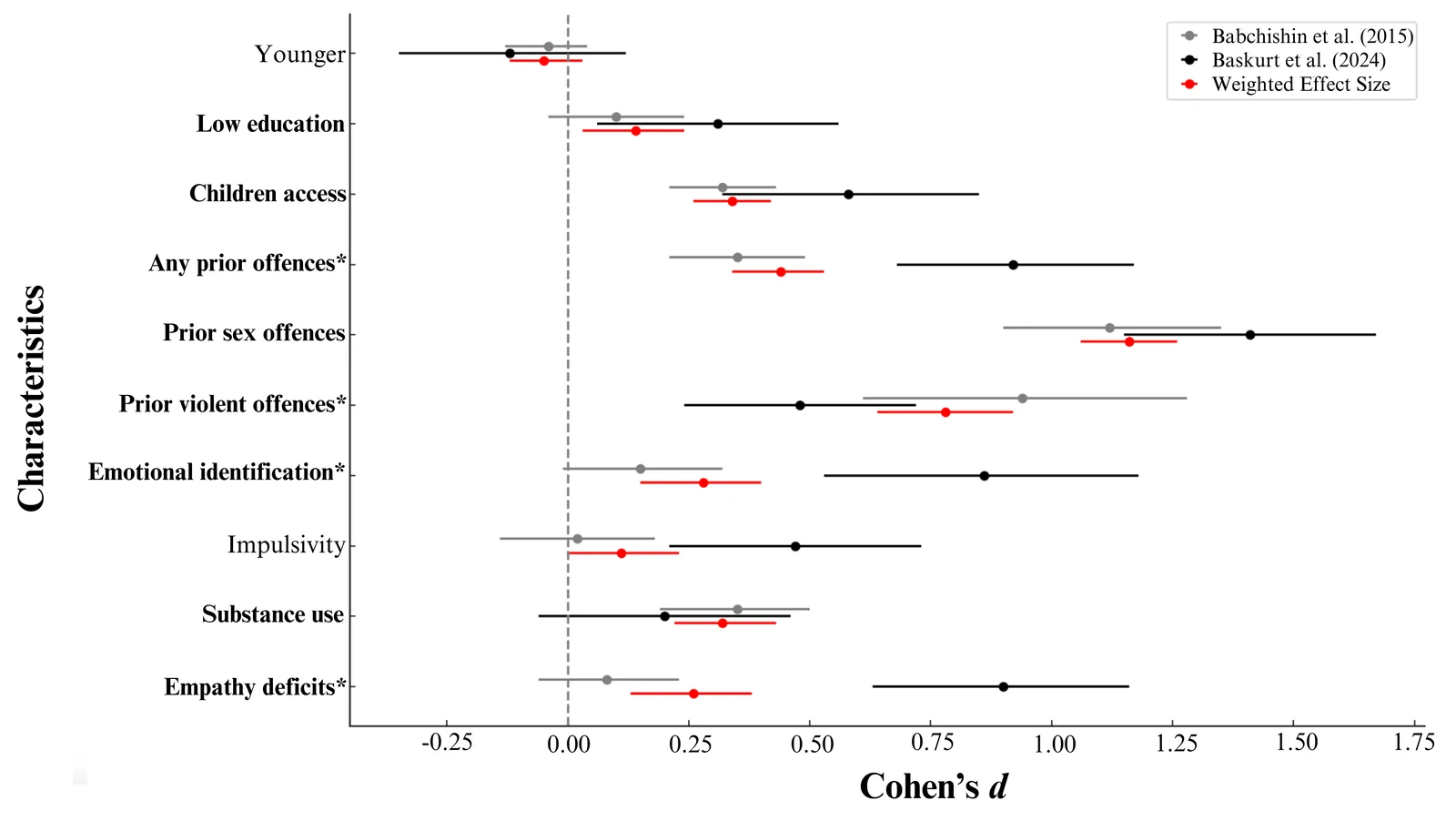
Forest Plot of Effect Sizes With 95% Confidence Intervals *Note.* Fixed-effect meta-analysis averages presented. The dotted black line at 0 on the X-axis indicates that CSEM-exclusive and mixed groups do not differ significantly (*p* < .05) on the variable. There are two different significance results displayed on the figure: bolded variables show *p* < .05 on *d_weighted_* (new meta-analysis), while those with an asterisk (*) show *p* < .05 on *Q*_Δ_.

Emotional identification with children displayed one of the most notable increases in effect size with the current study reporting *d* = .86 (95% CI [.53, 1.18]) and a weighted effect size of *d* = .28 (95% CI [.15, .40]). This change was statistically significant, as indicated by a value of 18.35 (*p* < .001; see Figure 1). These findings suggest a shift towards a stronger emotional identification with children among individuals with mixed offences.

Prior sex offences (*d_weighted_* = 1.16, 95% CI [1.06, 1.26]) showed an increase in effect sizes, however, the *Q*_Δ_ was not significant when comparing the new data against the old (*Q*_Δ_ = 4.03, *p* = .855). Any prior offences significantly increased (*d_weighted_* = .44, 95% CI [.34, .53], *Q*_Δ_ = 17.37, *p* = .026). The difference between both groups regarding prior violent offences showed a significant decreasing trend, though it was still large (*d_weighted_* = .78, 95% CI [.64, .92], *Q*_Δ_ = 9.06, *p* = .028).

### Indicators of Antisociality and General Psychological Profiles

There was no significant group difference between the two groups on empathy in the previous meta-analysis (*p* > .05; Babchishin et al., 2015). However, in the current cumulative meta-analysis, we found a significant effect size (*d_currentstudy_* = .90, 95% CI [.63, 1.16]; *d_weighted_* = .26, 95% CI [.13, .38]) and a meaningful change (*Q*_Δ_ = 29.05, *p* < .001; see Table 2 and Figure 1) indicating that these differences are now statistically significant, with CSEM-exclusive offending individuals showing more empathy compared to those with mixed offences.

Impulsivity also increased among the mixed group (*d_oldmeta_* = .02 versus *d_currentstudy_* = .47, *p* < .05), although this change was not statistically significant (*Q*_Δ_ = 9.26, *p* = .055). In the previous meta-analysis, substance use showed a significant effect (*d_oldmeta_* = .35, 95% CI [.19, .50], *p* < .05; see Table 2). However, we did not find any significant difference between both groups (*d_currentstudy_* = .20, 95% CI [-.06, .46], *p* = .997), and the difference between the previous meta-analysis and the current sample was not statistically significant (*Q*_Δ_ = 1.04, *p* = .905).

## Discussion

The purpose of the current study was to examine whether there have been any significant changes in individual characteristics since the former meta-analysis conducted by Babchishin et al. (2015). To explore shifting trends, we used the objective comparison of a cumulative meta-analysis (Hanson & Broom, 2005). Cumulative meta-analysis is especially useful in areas like this, where new primary data can be difficult to obtain, and where each new sample offers an opportunity to strengthen the evidence base. By combining our results with those of earlier studies, our findings provide a more stable and nuanced picture of the characteristics that distinguish CSEM-exclusive from mixed offending individuals. This approach helps clarify which differences between groups are consistent across studies and which may be sensitive to sample variation, information that is essential for informing clinical risk assessments, treatment planning, and prevention efforts.

We observed some changes in the psychological profiles of individuals involved in CSEM-related behaviours, particularly among those who have engaged in both online and contact forms of sexual harm. Compared to individuals involved exclusively in CSEM offences, those with a history of both CSEM and contact offending showed higher levels of emotional identification with children. This difference appears to have grown since the previous meta-analysis. Our findings are consistent with Paquette and Fortin (2023), who reported that individuals with mixed offence histories were nearly twice as likely to express cognitive patterns that frame children as romantic partners (11.5%, *n* = 26), compared to those with CSEM-only involvement (6.1%, *n* = 66). This kind of thinking may reflect a deeper emotional identification with children, which could increase the risk of both online and in-person offending.

We also found that general empathy deficits were more pronounced among individuals with mixed offence histories. This may be due, in part, to how empathy was measured in our study. Unlike earlier research that focused specifically on empathy toward victims (e.g., Babchishin et al., 2015), we used a broader measure of empathy that includes more general emotional and cognitive aspects. This approach may better capture the interpersonal and emotional functioning challenges that some individuals with mixed offence histories face. Emotional congruence reflects an individual’s distorted sense of psychological closeness to children–seeing them as companions or partners–without necessarily feeling concern for the child’s well-being (Finkelhor, 1984; Ward & Keenan, 1999). Meanwhile, general empathy deficits reflect broader interpersonal difficulties, which are not limited to the offence. Cognitive distortions, also referred to offence-supportive cognitions (e.g., minimizing harm or framing abuse as mutual) likely enable individuals to maintain reduced empathy for actual harm caused (Steel at al., 2020). Understanding this difference could help explain why mixed offending individuals display both higher emotional identification with children and lower general empathy.

Similar patterns were found by Yoon et al. (2025), who assessed both implicit and explicit measures of emotional congruence with children within 110 adult community males. They found that emotional congruence was moderately related to atypical sexual interests (*r* = 0.32 to 0.40, *p* < .001), particularly among individuals working with children. Yoon et al. (2025) also reported weak associations between self-reported emotional congruence and implicit positive evaluations of children (*r* = 0.17, *p* = .04) and no associations to general empathy, with the exception of distress in social interactions. Consistent with previous research (e.g., McPhail et al., 2018), these findings support the idea that emotional congruence with children and empathy are psychological processes, and that emotional congruence with children is generally unrelated to empathy. In the context of the current study, this distinction suggests that while mixed offending individuals may have greater emotional identification with children, this does not translate into broader empathic concern for others. This differentiation highlights the need to address both offence-specific cognitive distortions and general interpersonal functioning in risk assessment and treatment.

While it is possible that the online environment, through anonymity and shared communities, reinforces distorted thinking and emotional identification with children (Kloess & van der Bruggen, 2023), this does not fully explain the observed differences. Mixed CSEM offending tend to present with more complex psychological profiles (Babchishin et al., 2018). For example, prior studies suggest that they are more likely to show elevated levels of sexual preoccupation, difficulties with emotional intimacy, and cognitive distortions such as minimization and a lack of recognition of harm (Seto, 2019). These factors may help explain why individuals with mixed offending histories show both lower empathy and greater emotional alignment with children—risk factors that are associated with a broader and more persistent pattern of harmful behaviour (Mann et al., 2010; Seto et al., 2023).

### Limitations and Future Directions

The current study offers new perspectives on the evolving characteristics of those involved in CSEM offences, yet its contributions are bound by certain limitations. We used demographic information from the B.C. Corrections administrative data pull, along with items from the STABLE-2007 and ACUTE-2007 scales as indicators of offending characteristics, both of which have substantial research supporting their validity (e.g., Babchishin et al., 2023; Brankley et al., 2021; Nitsche et al., 2022). Although these items were scored based on participant interviews, it is important to acknowledge that only a single item was used per construct, which is suboptimal for ensuring reliability (Diamantopoulos et al., 2012). Ideally, multiple items would have been included to achieve a more robust measurement of each construct. Participants were scored multiple times on STABLE-2007 and ACUTE-2007 measures, and we only used their first assessment for their analyses. Additionally, the definition of the items in the current study also does not exactly match the definition of the items in Babchishin et al.’s (2015) meta-analysis (see Table S1, Supplementary Materials, for the definition of the items in the current study in comparison to Babchishin et al.’s [2015] study). An additional limitation is the unequal sample size between the offending groups (238 CSEM-exclusive versus 94 mixed), which may influence effect size estimates and reduce statistical power.

Another limitation of the current study is the data collection period. Data collection ranged from January 2005 and June 2013 (*Mdn* = 2009). Although this period is dated, it remains more recent than the data collection periods reported in Babchishin et al.’s (2015) meta-analysis. Among the six studies we used for the current cumulative meta-analysis (i.e., Finkelhor et al., 2009, 2012; Long et al., 2013; Neutze et al., 2011, 2012; Smid et al., 2015) that reported the data collection period, the dates ranged from June 2000 to August 2011 (*Mdn* = 2006). While our data are more recent than those in Babchishin et al. (2015), they were still collected between 2005 and 2013 (*Mdn* year = 2009) and thus may not reflect the most recent trends. Recent research suggests that the profile of individuals involved in CSEM offences may be evolving, with evidence indicating that individuals with CSEM offences are younger and that some offences may involve peer-to-peer dynamics, particularly in digital environments (Steel et al., 2024; van Wijk & Esseveldt, 2021). These trends suggest a possible shift in offender motivation, social context, and developmental background, potentially reflecting greater accessibility to exploitative material and the normalization of coercive behaviours in online spaces. While our study offers important insights, it is based on data collected between 2005 and 2013, and may not fully capture these newer developments. Ongoing research with more contemporary samples is needed to explore how age and online interaction patterns are reshaping offender typologies and risk profiles. An updated review or meta-analytic synthesis incorporating more recent data would help assess whether these apparent shifts represent meaningful changes in the population or continuity in underlying risk processes.

An essential aspect to consider is how CSEM-exclusive groups are defined in research. Some studies (e.g., Tomak et al., 2009), including the current study, define CSEM-exclusive group as those whose sexual criminal history is inclusively CSEM (excluding other non-contact offences). Other studies (e.g., Eke et al., 2019) offer more flexibility by allowing for the inclusion of non-contact sexual offences in CSEM-exclusive groups. Additionally, some studies (e.g., Merdian et al., 2018) categorize their offending groups based on self-report. Our study utilized administrative correctional data and so our classification was based on official records. To the extent that contact sexual offences are present in our CSEM-exclusive groups, our effect sizes may have been attenuated.

The current study also did not differentiate between individuals with exclusive pedophilic interests and those with teleiophilic or non-exclusive sexual interests. This lack of distinction restricts the specificity of our findings, particularly in interpreting the role of sexual preference in empathy deficits and emotional congruence with children. Prior research has indicated that sexual preference patterns may significantly mediate these psychological constructs (Oginni et al., 2022). Future studies should incorporate more refined measures of sexual interest to determine whether these factors differentially contribute to the observed empathy profiles.

### Practical Implications

Our findings indicate that differentiating individuals involved in CSEM offences by their criminal history, specifically whether they have committed contact sexual offences, offers a clearer understanding of the characteristics distinguishing the subgroups. Effective interventions and treatment programs should prioritize the identification of dynamic predictors of recidivism, for example, substance use or emotional identification with children, and recognize the importance of tailoring treatment intensity to align with individual risk levels (Bonta & Andrews, 2024). Consequently, with a comprehensive understanding of the unique characteristics that differentiate CSEM offending subgroups, treatment programs can better customize their interventions and offer tailored treatment. For instance, since mixed individuals showed greater impulsivity in the current study, treatment programs may prioritize targeting this trait amongst mixed individuals than amongst those with CSEM-exclusive offences.

While some characteristics between the two groups have remained consistent, others have changed. For example, empathy deficits and emotional identification with children, which initially showed non-significant effects, demonstrated significantly larger effects in this cumulative meta-analysis. Mixed offending individuals may therefore show greater empathy deficits and emotional identification with children than previously reported. Additionally, this cumulative meta-analysis revealed changes in any prior offences and prior violent offences, suggesting that the criminal history of mixed offending individuals may now be characterized by more overall offences, but fewer violent offences than previously reported. On the other hand, this cumulative meta-analysis revealed no significant changes in individuals’ age, education, victim access, prior sexual offences, impulsivity, or substance use compared to findings from the previous meta-analysis.

Identifying these shifts is also important for law enforcement strategies, particularly for case prioritization management. The current study suggests that mixed offending individuals are at a higher risk for sexual recidivism, given their heightened empathy deficits, impulsivity, and emotional connection to children – all recognized risk factors for sexual recidivism (Babchishin et al., 2023; Mann et al., 2010; Seto et al., 2023). Cases involving mixed offending individuals may therefore be flagged as high priority for investigation and intervention to enhance resource allocation. Individuals with a criminal history characterized by more overall offences and violent offences, along with greater empathy deficits and emotional identification with children, may also be those more likely to engage in mixed offending. In short, it is crucial to adapt our risk assessments and treatments continuously, reflecting the changing profiles of those involved in CSEM offences.

## Supplementary Materials

The online supplement includes:

1. Table S1, a table comparing the definitions used in the former meta-analysis (Babchishin et al., 2015) and the current meta-analysis (Baskurt et al., 2026S-a); and
2. The R code for our cumulative meta-analysis code (Baskurt et al., 2026S-b).

BaskurtS.
O’DonaghyM.
BabchishinK. M.
 (2026S-a). Supplementary materials to "Understanding variations among individuals with child sexual exploitation material offences: A cumulative meta-analysis"
[Table S1]. PsychOpen. 10.23668/psycharchives.21792
PMC1361014442788147

BaskurtS.
O’DonaghyM.
BabchishinK. M.
 (2026S-b). Supplementary materials to "Understanding variations among individuals with child sexual exploitation material offences: A cumulative meta-analysis"
[R code]. PsychOpen. 10.23668/psycharchives.21793
PMC1361014442788147

## Data Availability

The data that support the findings of this study are available from BC Corrections, but restrictions apply to the availability of these data, which were used under license for the current study, and so are not publicly available. Data are, however, available from the authors upon reasonable request and with permission of BC Corrections. Please email the corresponding author for additional details.

## Social Media Accounts

*Kelly M. Babchishin:* X
